# Editorial to “Ischemic stroke associated with high grade pedunculated device related thrombosis following left atrial appendage closure”

**DOI:** 10.1002/joa3.13086

**Published:** 2024-05-25

**Authors:** Wei‐Ta Chen

**Affiliations:** ^1^ Division of Cardiology, Department of Internal Medicine Taipei Medical University Hospital Taipei Taiwan; ^2^ Department of Internal Medicine, College of Medicine, School of Medicine Taipei Medical University Taipei Taiwan

In this issue, Chatani Ryuki presented a case entitled “Ischemic stroke associated with high grade pedunculated device related thrombosis following left atrial appendage closure.”^1^ The authors presented a case with atrial fibrillation and stroke despite on oral anticoagulation therapy (OAC). They performed left atrial appendage closure (LAAC) with Watchman (Boston Scientific) and discontinued OAC. However, device related thrombus (DRT) was noted at 314 days after LAAC. DRT was successfully resolved by OAC and OAC was kept as long‐term medication. In the presented case, the post‐LAAC drug regimen and the happening of DRT deserve more discussion.

LAAC was considered a reasonable alternative option to OAC in atrial fibrillation. In 2015, the first LAAC device (Watchman, Boston Scientific) was approved by Food and Drug Administration of United states. After that, patients with atrial fibrillation and CHA2DS2‐VAS score higher than^2^ have an option to take LAAC if they have contraindications for OAC. In the consensus statements about LAAC from major societies (Heart Rhythm Society, Asia‐Pacific Heart Rhythm Society and European Heart Rhythm Association), all listed LAAC in patients contraindicated to OAC as Class I indication. Based on the current available evidences, there is almost no doubt that LAAC should be taken if one is unable to take long‐term OAC.

However, there are some uncertainty in the other indications of LAAC. Can LAAC replace OAC even in patients without contraindication to OAC? This is one of most often discussed questions. The role of OAC for stroke prevention in atrial fibrillation has long been established based on many large‐scale clinical trials. On the other hand, trials comparing LAAC and OAC were much less. PRAGUE‐17 trial is the current largest prospective trial comparing LAAC and OAC.^2^ It enrolled 402 patients with 50% receiving LAAC and 50% taking OAC. After 1 year, the stroke rate and bleeding rate were similar in both groups. However, after 4 years, the LAAC group was with lower composite risk rates (including stroke, transient ischemic attack, systemic embolism, bleeding, and cardiovascular death) than the OAC group. While the stroke rate is similar in both groups, the major difference of composite risk came from the lower rate of non‐procedural bleeding risk of LAAC group.

Based on this finding, LAAC may offer patients a benefit at reduction of bleeding, rather than at a stronger protection from stroke than OAC. Such finding echoes the suggestion from the consensus statements of the societies. Due to the lack of stronger protection of LAAC, OAC is still the main suggestion for patients with high CHA2DS2‐VAS score. For those with high bleeding risk, LAAC may be a reasonable alternative to OAC.

To extend the concept, it affects the choice of drugs after LAAC, especially for those had stroke despite on OAC. For patients with contraindication to OAC, short‐term antiplatelet therapy should be used after LAAC. The purpose of using antiplatelet is to avoid thrombus formation before the endothelization of the LAAC. In other words, stroke was prevented predominantly by the occluded left atrial appendage, rather than by the drugs after LAAC. However, for patients with stroke despite on OAC, there was no clear suggestion about the drugs after LAAC. In this group of patients, OAC provides not enough protection from stroke. However, evidence did not prove LAAC more effective in stroke prevention than OAC. Therefore, for those who have stroke on OAC, LAAC alone may not be enough. Although lacking of direct evidence, keeping OAC even after LAAC for those with stroke on OAC is a reasonable and logical management.

Back to the presented case, the author stopped OAC after LAAC, even the patient had two systemic embolism episodes on OAC. After 314 days of LAAC, the patient experienced another ischemic stroke episode with the happening of DRT. If the patient was prescribed long‐term OAC after LAAC, the story may be completely different.

DRT is another issue to be addressed. Both Amulet IDE trial and SWISS APERO trial revealed similar DRT rates in Amulet (Abbott) and Watchman (Boston Scientific)^3^, ^4^ However, the timing of DRT may differ in different types of LAAC. In Amulet, majority of DRTs were happened early (<45 days post implantation). In Watchman, majority of DRTs ere happened later than 45 days. In this presented case, Watchman FLX was used, and the DRT was noted at 314 days after implantation. The finding was therefore compatible with the trials results.

Finally, in February 2023, a propensity scoring matching study comparing LAAC and OAC disclosed lower mortality rate and stroke/systemic embolism rate in the LAAC group.^5^ The bleeding risk is higher in LAAC group early after implantation, but lower from the sixth weeks after implantation. Based on the results of this study, the indication of LAAC may be even broaden to replace OAC in more situations. The study is published after the most recent consensus statements from the major societies. However, a single study may not offer strong evidence enough to alter the consensus statements. More studies are necessary to examine the role of LAAC and OAC in the future.

## FUNDING INFORMATION

N/A.

## CONFLICT OF INTEREST STATEMENT

None.

## ETHICS STATEMENT

N/A.

## Data Availability

N/A.
